# EEG‐Derived Index Predicts Postoperative Delirium in Elderly Patients With Hip Fracture: A Prospective Study From a Tertiary Medical Center

**DOI:** 10.1002/brb3.71218

**Published:** 2026-01-19

**Authors:** Ayixia Nawan, Geng Wang

**Affiliations:** ^1^ Department of Anesthesiology, Beijing Jishuitan Hospital Capital Medical University Beijing China

**Keywords:** electroencephalography, delirium index, elderly patients, hip fracture, postoperative delirium, POD prediction model

## Abstract

**Introduction:**

Postoperative delirium (POD) remains challenging to predict in elderly hip fracture patients. This study posited that a newly developed electroencephalogram‐derived metric, termed the Delirium Index (DELi), has the potential to elucidate latent information pertaining to the preoperative predisposing factors of POD.

**Methods:**

A prospective cohort of 144 elderly patients undergoing hip fracture surgery was enrolled. DELi scores were derived from preoperative electroencephalography (EEG) using wavelet analysis. POD was diagnosed using the Confusion Assessment Method (CAM). Predictive performance was evaluated via ROC analysis, logistic regression, and bootstrapping validation.

**Results:**

DELi demonstrated strong correlation with CAM scores (*r* = 0.516, *p* < 0.0001) and good predictive accuracy for POD (AUC = 0.791, 95% CI: 0.715–0.867). A composite model integrating DELi, Montreal Cognitive Assessment (MoCA), and FRAIL scores achieved excellent discrimination (AUC = 0.922, 95% CI: 0.878–0.965), with 85.9% sensitivity and 86.3% specificity.

**Conclusions:**

The DELi index, combined with cognitive and frailty assessments, provides a practical tool for preoperative POD risk stratification in elderly hip fracture patients.

**Trial Registration:**

ChiCTR2200060389

## Graphical abstract

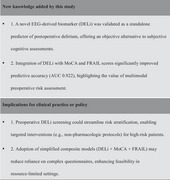

## 1 Introduction

As the world's population is aging rapidly, perioperative management in the elderly has become a public health topic of high interest. Postoperative delirium (POD) constitutes the predominant complication in the postoperative period among elderly patients, ranking as a significant menace to healthcare provision (Rudolph and Marcantonio 2011). Delirium is a complex neuropsychiatric syndrome present as hyperactivity, hypoactivity, or a combination of both, reflecting the diverse clinical presentations of delirium (Krogseth et al. 2011). In elderly patients, due to the complexity of perioperative management, auxiliary assessments beyond scale assessments may guide the implementation of multimodal preventive delirium treatment protocols in clinical guidelines to reduce the severity and duration of delirium in patients. Traditional models for predicting postoperative delirium, like those used for cardiac surgery, often perform poorly, with a pooled AUC of only 0.60 (Wueest et al. 2023). This highlights the need for improved models that use more accessible data and advanced analytics. The Delirium Observation Screening (DOS) scale helps in early detection and intervention of delirium, potentially minimizing its negative effects (Park et al. 2025). Recognizing risk factors like advanced age, cognitive impairment, and intraoperative hyperglycemia can also assist in predicting and preventing delirium (Koster et al. 2009).

Machine learning offers a solution, as demonstrated by a study that used continuous physiological data to predict ICU delirium, achieving AUCs of 0.82 and 0.84 in internal and external validations, respectively (Windmann et al. 2019). The timing of data gathering is vital for POD due to the substantial correlation between pre‐existing and triggering factors (Shih et al. 2025; Lindroth et al. 2018; Zhang et al. 2019; Zhao et al. 2021). This limitation is significant because intraoperative data, while potentially rich in predictive value, are not always readily accessible or consistently recorded across different healthcare settings. The short gap between data collection and POD evaluation could potentially inflate the model's performance and undermine its clinical usefulness (Inouye 1996). A more logical and practical approach would be a streamlined model that only uses data obtained before the surgeries (Shih et al. 2025).

In recent years, it has been shown that changes in cognition and emotion can cause significant changes in electroencephalography (EEG) and that EEG has the potential to be an indicator reflecting pain extent, cognitive function, and emotional state (Kim et al. 2016; Nir et al. 2010). In this study, the delirium index (DELi, ranging from 0–100) was collected following the entire frequency band EEG wavelet algorithm as a novel risk prediction indicator for the early detection of MCI and acute confusion of cognition (delirium) (Dauwels et al. 2009). We hypothesized that the DELi score could also extract associated attributes from predisposing factors of POD (Wu 2017) and at least partly summarize these factors when collected preoperatively. Existing models often exhibit AUCs <0.791 and rely on intraoperative data which is difficult to obtain (Hayhurst et al. 2016; Wang et al. 2017; Yang et al. 2016; Liang et al. 2015; Dworkin et al. 2016). By quantifying brain state and integrating multifactorial aspects (e.g., diminished cognitive reserve), DELi offers a more objective measure than traditional scales, particularly advantageous in patients with communication barriers.

The principal aim of this study was to assess the validity of DELi as an independent preoperative predictor of POD. The secondary aims included the development of a parsimonious predictive model by integrating DELi with clinical indicators, as well as the evaluation of the clinical feasibility of this approach.

Given that delirium prediction models are advised to target specific populations, (Shih et al. 2025) and POD is prevalent among elderly patients undergoing hip fracture repair surgeries—with reported prevalence rates ranging from 36.5% to 53.3%—this study specifically included elderly hip fracture patients (Rudolph and Marcantonio 2011; Korc‐Grodzicki et al. 2015; Inouye et al. 2014; Aldecoa et al. 2017).

## 2 Methods

### 2.1 Study Design

**FIGURE 1:**
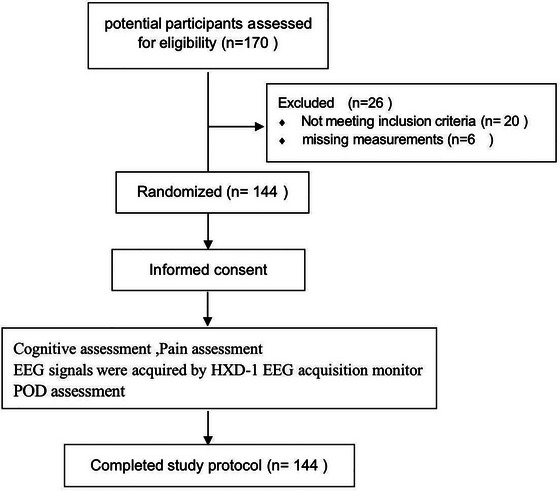
Flow diagram of the study protocol.

This study is a prospective longitudinal cohort study, including elderly hip fracture patients treated at Jishuitan Hospital, all of whom signed informed consent forms. The flowchart of the study protocol is shown in Figure 1.

### 2.2 Participants

The participants had the following characteristics: age ≥ 65 years, admission to the geriatric orthopedic ward, and American Society of Anesthesiologists (ASA) classification I–III. Exclusion criteria were self‐withdrawal, canceled surgery, illiteracy and visual impairment, long‐term chronic analgesic drug users, history of alcohol abuse, unavailable patient EEG monitoring data, and participation in other clinical trials within 30 days. Only the patients lined up for the day's first procedure were considered, in order to reduce the possible prejudice brought about by circadian rhythms (Sieber et al. 2018).

All patients received ultrasound‐guided fascia iliaca compartment block and routine spinal anesthesia. Routine intravenous patient‐controlled analgesia was used for all.

### 2.3 Outcome

The Confusion Assessment Method (CAM) was employed to diagnose postoperative delirium in patients (Korc‐Grodzicki et al. 2015; Coppola et al. 2020). On the first and second days postoperatively, a trained geriatrician visited twice daily (08:00 and 20:00) to assess the presence of POD. POD was diagnosed if delirium was identified based on the presence of both criteria 1 and 2, along with either criterion 3 or 4, during any of the assessments.

### 2.4 Predictors

DELi scores were collected from a wavelet algorithm multifunction monitor (Beijing Easymonitor Technology Co., Ltd., Beijing, China) prior to anesthesia in operation rooms (see Supplemental Figure S1). The sensor gathers EEG signals from the brain's prefrontal lobe every 2 min continuously for 20 min and sends them to a preprocessing circuit. The system amplifies, filters, and converts these signals to digital form. It segments the EEG waves into windows and uses wavelet analysis to extract characteristic metadata. A multiple regression algorithm identifies metadata linked to brain function outcomes. After weighted normalization, feature index groups are created.

Patients remained awake and calm without undergoing any procedures or using any sedative or antiemetic drugs until the DELi scores were collected (see Supplemental Calculating DELi Scores).

In addition, demographic and clinical characteristics of patients were collected preoperatively, including age, sex, fracture type, surgical procedure, time interval between fracture and surgery, cognitive function, and frailty status, and postoperative complications (e.g., cardiovascular events, respiratory failure, deep vein thrombosis, pulmonary embolism, acute kidney injury, pulmonary embolism, sepsis, or reoperation). Cognitive function was assessed using the Montreal Cognitive Assessment (MoCA, 0 to 30 points, with ≥ 26 suggesting cognitive deficiencies). (Palanca et al. 2017) Frailty status was assessed using the FRAIL scale (0 to 5 points, with 0 denoting robust condition and ≥ 3 denoting frail condition) (Trzepacz et al. 2015).

### 2.5 Sample Size

Our pretest showed the DELi score had 79% sensitivity and 80% specificity for detecting POD, with a 50% incidence rate. To achieve a two‐sided 95% confidence interval (CI) with a maximum width of 0.20 for sensitivity, a sample size of 128 is needed, and for specificity, 124 is required. Accounting for an 11% dropout rate, 144 patients are needed to confirm POD discrimination.

### 2.6 Statistical Analysis

Missing data were addressed with multiple imputations. Continuous and categorical data were expressed as median with IQR or mean with SD and number with proportion. Continuous and categorical data were represented by the mean with standard deviation (SD) or median with interquartile range (IQR) and number with proportion, respectively. The area under the ROC curve (AUC), sensitivity, and specificity were determined using ROC curve analysis. The optimal cut‐off value was identified by maximizing the Youden index. (Aprahamian et al. 2017) AUC was used to assess the discrimination, while the calibration curve was plotted and evaluated with the Hosmer–Lemeshow test. The clinical usefulness was assessed by decision curve analysis (DCA). The bootstrapping method (resampling = 1000) was employed for internal validation. Statistical analysis was performed using R software (version 4.2.0, R Foundation for Statistical Computing, Vienna, Austria; https://www.R‐project.org), with *p* < 0.05 considered statistically significant.

## 3 Results

### 3.1 Clinical Characteristics

A total of 170 patients were approached, of whom 144 qualified for the analysis (Figure 1). Table 1 presents the demographic and clinical characteristics of the patient cohort, with a median age of 78.0 years (interquartile range [IQR]: 71.0–85.8) and 38.2% (*n* = 55) males. POD assessments were successfully conducted in all participants, with 71 patients (49.3%) identified as POD. Logistic regression analysis revealed a significant association between delirium and age (odds ratio [OR] = 1.066; 95% confidence interval [CI], 1.010–1.126), as well as the MoCA score (OR = 0.375; 95% CI, 0.255–0.551). The patients did not report any significant discomfort associated with the acquisition of DELi scores. Additionally, no severe postoperative complications were observed in either group. No data were missing.

**TABLE 1:** Baseline demographic and clinical characteristics of participants.

	POD	No POD	*p*‐value
	*n* = 71	*n* = 73	
Age (years)a(years)	82.0 [75.0, 88.0]	76.0 [69.5, 83.0]	< 0.001
Sex (male/female)a	32/39	23/50	0.094
MoCA (IQR)b	25.0 [24.0, 27.0]	27.0 [27.0, 28.0]	< 0.001
Type of fracturea			0.248
Femoral neck fractures	39	47	
Intertrochanteric fractures	32	26	
Surgical procedurea			0.843
Cannulated screw fixation	1	1	
THA	7	7	
PFNA	32	28	
Hemi‐arthroplasty	31	37	
Time interval between fracture and the operationb(h)	25.0 [20.0, 40.0]	36.0 [22.0, 40.0]	0.070
Hospital stayb(days)	5.0 [4.0, 6.0]	5.0 [4.0, 5.0]	0.054
FRAIL score (IQR)	3 [2, 3]	1 [1, 2]	< 0.001
DELi score (IQR)	49 [41, 59]	28 [19, 35]	< 0.001

**Abbreviations**: IQR, interquartile range; MoCA, Montreal Cognitive Assessment; PFNA, proximal femoral nail anti‐rotation; POD, postoperative delirium; THA, total hip arthroplasty.

Data are shown as n (%) or median [IQR].

### 3.2 Correlation Between DELi and CAM Score

**FIGURE 2:**
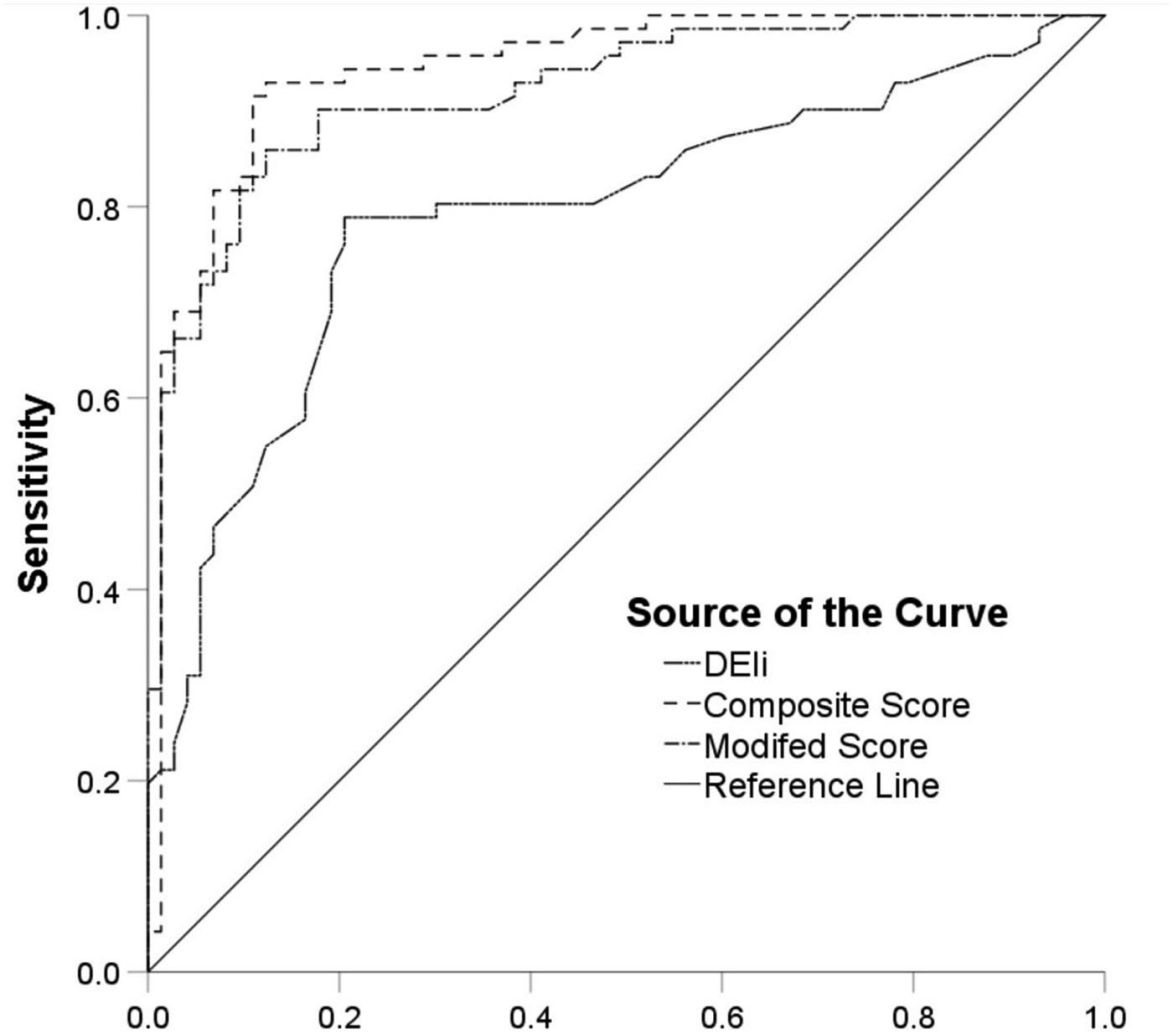
ROC curve analysis of DELi and CAM scores.DELi curve: AUC = 0.791, 95% CI [0.715–0.867]; Composite score AUC = 0.944, 95% CI [0.907–0.982]; Modified composite score, AUC = 0.922, 95% CI [0.878–0.965]. **Abbreviations**: AUC, area under the curve; ROC, receiver operating characteristic.

DELi is moderately correlated with CAM scores (*r* = 0.516, *p* < 0.0001) (Table 2). The largest Youden's index of the DELi was 39.5, which was used as the cutoff for predicting POD. The predictive sensitivity and specificity of DELi for POD were 78.9% (with a 95% confidence interval from 69.4% to 88.4%) and 79.5% (with a 95% confidence interval from 70.2% to 88.7%), respectively, yielding a diagnostic accuracy of 79.2% (with a 95% confidence interval from 72.5% to 85.8%). Using the Hosmer and Lemeshow test (*p* = 0.054), the accredited calibration was confirmed. The discrimination for predicting POD was evaluated by using ROC (Figure 2), with AUC = 0.791 (95% CI: 0.715–0.867). (Figure 2, DELi curve).

**TABLE 2:** Contingency table of the DELi score by POD.

DELi score	POD		
	Positive	Negative	Total
Positive	56	15	71
Negative	15	58	73
Total	71	73	144

**Abbreviations**: DELi, delirium index; POD, postoperative delirium.

### 3.3 Composite Score of DELi

To develop a composite score, a backward logistic regression for predicting POD was used. In addition to the DELi score, each baseline demographic and clinical characteristic of participants was used as a candidate for fitting the model. The DELi score, surgery, MoCA, and frailty index were entered into the final model. The AUC for predicting POD was 0.944 (95% CI: 0.907–0.982). (Figure 2 Composite score curve).

### 3.4 Modified Composite Score of DELi

To develop a more adaptable score, only DELi, MoCA, and frailty scores were used to develop a modified composite score. (Table 3)

**TABLE 3:** Logistic regression analysis of the risk factors for POD.

	POD n = 71	Non‐POD n = 73	Regression coefficient	*p*	OR (95% CI)
**DELi**	49 [41, 59]	28 [19, 35]	0.065	0.000	1.067 1.032–1.104
**MoCA**	25 [24, 27]	27 [27, 28]	−0.602	0.002	0.547 0.371‐0.807
**FRAIL_Score**	3 [2, 3]	1 [1, 2]	1.389	0.000	4.010 2.185–7.358

**Abbreviations**:CI, confidence interval; DELi, delirium index; IQR, interquartile range; MoCA, Montreal Cognitive Assessment; OR, odds ratio; POD, postoperative delirium.

Data are shown as the median [IQR].

The formula for a modified composite score is as follows:

$$\mathrm{Modified}\;\mathrm{score}=\frac{(\mathrm{EXP}\;\left(10.534+0.065\times \;\mathrm{DELi}\;-0.602\times \;\mathrm{MoCA}\;+1.389\times \;\mathrm{Frailty}\;\mathrm{index}\right)}{1+(\mathrm{EXP}\;\left(10.534+0.065\times \;\mathrm{DELi}\;-0.602\times \;\mathrm{MoCA}\;+1.389\times \;\mathrm{Frailty}\;\mathrm{index}\right)}$$

The AUC of this modified composite score for predicting POD was 0.922 (95% CI: 0.878, 0.965). (Figure 2 Modified score curve). The Hosmer and Lemeshow test (*p* = 0.683) (Supplementary Figure S2) was used to verify the certified calibration.

The largest Youden's index of the DELi was 0.48, which was used as the cutoff for predicting POD. An increased concordance between DELi and CAM scores was yielded (Table 4). The modified composite score's sensitivity and specificity for forecasting POD were individually 0.859 (95% CI 0.778‐0.94) and 0.863 (95% CI 0.784–0.942), with a diagnostic precision of 0.861 (95% CI 0.805–0.912).

**TABLE 4:** Contingency table of the modified composite score by the POD.

DELi score	POD		
	Positive	Negative	Total
Positive	61	10	71
Negative	10	63	73
Total	71	73	144

**Abbreviations**: DELi, delirium index; POD, postoperative delirium.

### 3.5 Adverse Events

No significant adverse events occurred as a result of DEli_Score testing. Between patients with and without POD, the length of stay had no significant difference (*p* = 0.054).

## 4 Discussion

A novel EEG‐derived delirium‐associated index, the DELi score, was introduced, which could be used for predicting POD in elderly patients undergoing hip fracture repair surgery. The results showed the analysis of ROC curves for 144 paired values revealed that DELi had a good predictive accuracy for the presence of POD (Figure 2). The optimal cutoff value of DELi for predicting delirium occurrence was 39.5 (in which the sensitivity, specificity, and Youden index were 0.789, 0.795, and 0.584, respectively) (Figure 2, DELi curve).

At present, there is no objective, reliable, quantitative, and advanced simple way that can measure cognition level within clinical practice and predict POD. Subjective assessments are highly demanding for assessors. Unlike commonly applied behavioral assessments, the prediction model is based on physiological signals, being more direct than behavior.

Although it is commonly assumed that patients with delirium have a longer hospital stay, in the present study, we found no significant difference in length of stay between the two groups of patients (with and without postoperative delirium) (*p* = 0.054). Under the enhanced recovery after surgery guidelines of local health centers, elderly patients with hip fractures are often discharged on the second to fourth day after surgery. Within our study cohort, to achieve consistency in follow‐up periods, POD was examined only on the first two days after surgery for every patient and exhibited a high incidence of POD at 49.3%; the incidence rates of delirium were substantially elevated in patients exhibiting cognitive impairment, as compared to their counterparts without such deficits, and demonstrated a positive correlation with advancing age. (see Table 1).

Standardization of delirium assessment and ideal POD prevention strategies during the perioperative period are essential, as postoperative identification undoubtedly delays intervention, attenuating effectiveness. A variable that directly quantifies brain status and extracts pertinent attributes from a multitude of interacting preoperative risk factors might facilitate the development of a concise predictive model.

EEG has been utilized in delirium research for several decades (Youden 1950). The DELi score was developed from the whole frequency band EEG wavelet algorithm (Engel and Romano 2004; Sun et al. 2021). When delirium was present, reproducible and consistent alterations were identified in brain waves, serving as a characteristic indicator of delirium (Dauwels et al. 2009). We hypothesized that the DELi score could also extract associated attributes from predisposing factors of POD and at least partly summarize these factors when collected preoperatively.

Numerous susceptibility and precipitating risk factors have been associated with POD, such as advanced age, increased comorbidities, preoperative cognitive impairment, benzodiazepines, opioids, hypotension, intraoperative blood loss, and insufficient pain management (An et al. 2017; Neuman et al. 2019; Rengel et al. 2018). Several POD prediction tools have been developed, containing complex and numerous predictive factors, with discriminative abilities ranging from acceptable (AUC from 0.638 to 0.814) (Lindroth et al. 2018; Zhang et al. 2019; Yang et al. 2016; Liang et al. 2015; Dworkin et al. 2016). However, these models typically incorporate intraoperative and postoperative data, such as the use of vasopressors during surgery, transfusions, surgical and anesthesia durations, and postoperative intensive care data (Lindroth et al. 2018; Zhang et al. 2019; Yang et al. 2016). Our findings identified factors such as age, MoCA score, fracture, and time between fracture and surgery as significantly associated with postoperative delirium, consistent with previous research, indicating the importance of these factors in the occurrence of postoperative delirium in elderly patients. This striking distinction suggests that under sufficiently adverse conditions of precipitating factors, notably surgery, it is easy for this susceptible population to develop POD.

Our analysis elucidated a substantial correlation between the duration from fracture to surgical intervention and the onset of delirium. (see Table 2). The timing window between data collection and POD assessment is crucial and imperative for predicting POD, given the significant interplay between prodromal and precipitating factors (Shih et al. 2025; Korc‐Grodzicki et al. 2015). A narrow time interval might exaggerate the performance of assessment tools and compromise their clinical utility, for instance, ICU admission possibly occurring post‐delirium onset, even suggesting overlap between data collection and POD assessment. An optimal strategy for the prevention of postoperative delirium (POD) should be implemented perioperatively, and discrimination after surgery delays the intervention undoubtedly and attenuates the effects. Therefore, DELi scores might replace the numerous factors influencing susceptibility to POD necessitating the exclusion of many intricate questionnaire surveys that were previously deemed essential in earlier models. (Lindroth et al. 2018, Zhang et al. 2019, Yang et al. 2016, Liang et al. 2015, Dworkin et al. 2016) Meanwhile, in our study, the DELi scores alone exhibited comparable or even superior discrimination in predicting POD (AUC = 0.791). The majority of them are complex delirium risk prediction models with heterogeneous variables and definitions. (Shih et al. 2025)

More complex questionnaires and cautious reviews may lead to better predictions but deviate from clinical practice. Models with too many predictive factors also risk overfitting, especially when the predictive factors may be significantly correlated. Integrating these factors with DELi can construct a more accurate prediction model, further enhancing prediction accuracy and reliability. Combining DELi scores with a few predictive factors derived from prospective longitudinal cohorts of relevant precipitating factors could facilitate the development of a concise model with few predictive factors. When clinical characteristics of the patients (surgery type, delirium index score, MoCA, and frailty score) were placed into the model, we obtained a combined score with AUC values that reached 0.944. Due to that both are associated with increased comorbidity and mortality, cognitive impairment and frailty are two perilous clinical conditions for the elderly (Weiss et al. 2023). Similarly, only DELi, MoCA and frailty scores were added to develop the revised composite score to improve the practicability (Figure 2 Modified score curve). The AUC value of the score was 0.922 with excellent discrimination, the sensitivity was higher than 85%, and the accuracy increased. (Table 4). Conventionally, AUC values > 0.90 are considered to represent excellent discrimination. (Chong et al. 2015). Notably, the wide spectrum of threshold probabilities, ranging from 5% to 95%, underscores its potential value as a promising tool for guiding clinical decisions. Furthermore, comparative analysis revealed that constructing a new model using binary DELi scores did not significantly alter predictive accuracy, further validating the effectiveness and stability of the delirium index.

Delirium is multifactorial, necessitating personalized, multicomponent preventive and control strategies to mitigate its incidence. Regardless of the delirium phenotypes, all of them had an association with long‐term cognitive impairment (Flach et al. 2011). Using this DELi index for decision‐making yielded more net benefits. POD can be predicted by experienced clinical physicians. However, the ambiguity of delirium risk may increase when the risk level is moderate or when clinical experience is limited. In particular, low‐activity subtype delirium is often misdiagnosed as depression or fatigue in elderly patients (Girard et al. 2018; Gan 2017).

It is noteworthy that this index was developed for elderly undergoing hip fracture surgery, where POD is particularly prevalent. Therefore, it may not be generalized to other populations. Of greater importance, we can establish a concise and excellent model using some preoperative measurements. Sensitivity analysis also showed that using binary DELi classification instead of continuous DELi scores did not change the model's discriminative ability. This suggests that DELi scores, or their binary classification, can serve as robust predictors or contribute to the model when predicting POD. Nonetheless, it is imperative for researchers to extend these findings to novel populations and substantiate them through comprehensive external validation.

### 4.1 Limitations

Although the study results contribute to understanding cognitive status during the perioperative period in elderly patients, there are several limitations. A detailed assessment of comorbidities in patients aged 80 and above was not conducted; future research should extend current findings to explore more potential interactions. The single‐center study was conducted with a limited number of samples. Nevertheless, the sample size fulfilled the criteria of our statistical hypotheses. Furthermore, data collection was limited to postoperative days 1 and 2, which precluded the assessment of long‐term outcomes. The findings of this study are pertinent to elderly patients undergoing orthopedic surgery. Moreover, the study utilized all available data for model development, conducting only internal validation. This approach may result in an overly optimistic assessment of model performance. Therefore, further research is necessary. Additionally, it's important to note that the sample size was determined for predicting POD, so the variation in hospital stay length between patients with and without POD was not found because of insufficient power. It remains to be determined whether they can be generalized to other populations (i.e., age‐matched healthy older adults and elderly patients in inpatient and outpatient settings).

## 5 Conclusion

A new EEG‐based index, known as the DELi score, has the potential to predict POD in older individuals having hip fracture repair procedures. It may represent an important basis for developing clinical treatment plans and multimodal rehabilitation protocols and reducing major postoperative complications.

## Author Contributions

A. N. contributed to data generation, collection, analysis, interpretation, and the conception and writing of the manuscript. G. W. contributed to data generation, collection, and writing of the manuscript. All authors had full access to the data, contributed to the study, approved the final version for publication, and take responsibility for its accuracy and integrity.

## Conflicts of Interest

The authors declare no conflicts of interest.

## Funding

The authors have nothing to report.

## Ethics Statement

This study was approved by the Institutional Review Board of Beijing Jishuitan Hospital (approval number: 202102–11).

## Consent

Patient consent is not required because no personal information or details are included that may identify the patient.

## Declaration

This research has not been presented or published.

## Clinical Trial Number and Registry Uniform Resource Locator

The methodology of data collection was registered before the enrollment of patients at the China Clinical Trials Registry Platform (ChiCTR2200060389).

## Supporting information




**Supplementary Material**: brb371218‐sup‐0001‐SuppMat.docx

## Data Availability

The data that support the findings of this study are available from the corresponding author, Ayixia Nawan upon reasonable request.
