# Supplementary material for: EEG‐Derived Index Predicts Postoperative Delirium in Elderly Patients With Hip Fracture: A Prospective Study From a Tertiary Medical Center
Source: Brain Behav. 2026 Jan 19;16(1):e71218. doi: 10.1002/brb3.71218 (PMC12816766; doi:10.1002/brb3.71218)
Supplement: Supplementary file 1 — Supplementary Material: brb371218‐sup‐0001‐SuppMat.docx [file BRB3-16-e71218-s001.docx]

**Legends for Supplementary Figures**

**
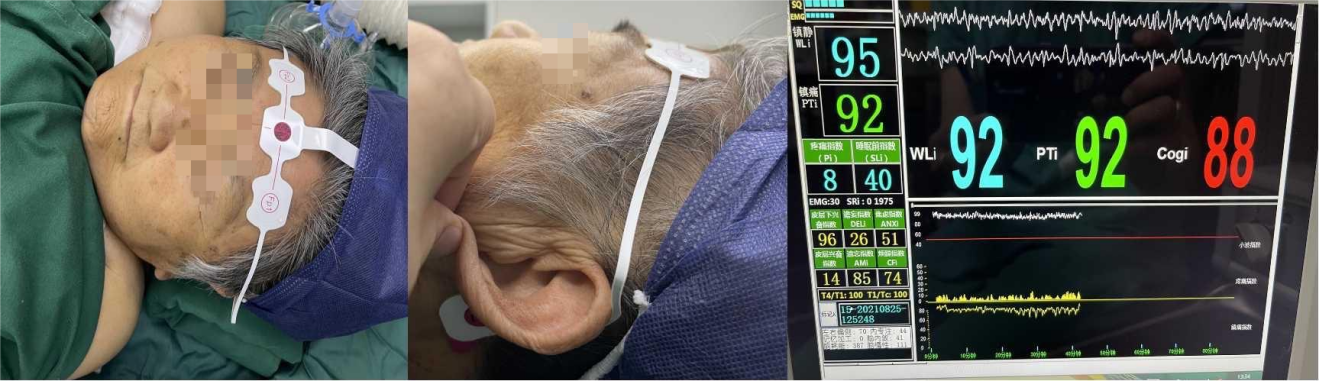
**

**Supplemental Figure 1.** Placement of EEG electrodes and display of DELi. (a) Three on the forehead of each subject; (b) Two reference electrodes were placed on the mastoid behind the ears bilaterally (the left one is shown here); (c) DELi was displayed in real-time on an integrated EEG monitor, with values updated every 2 s (sampling rate: 1600 Hz).

**
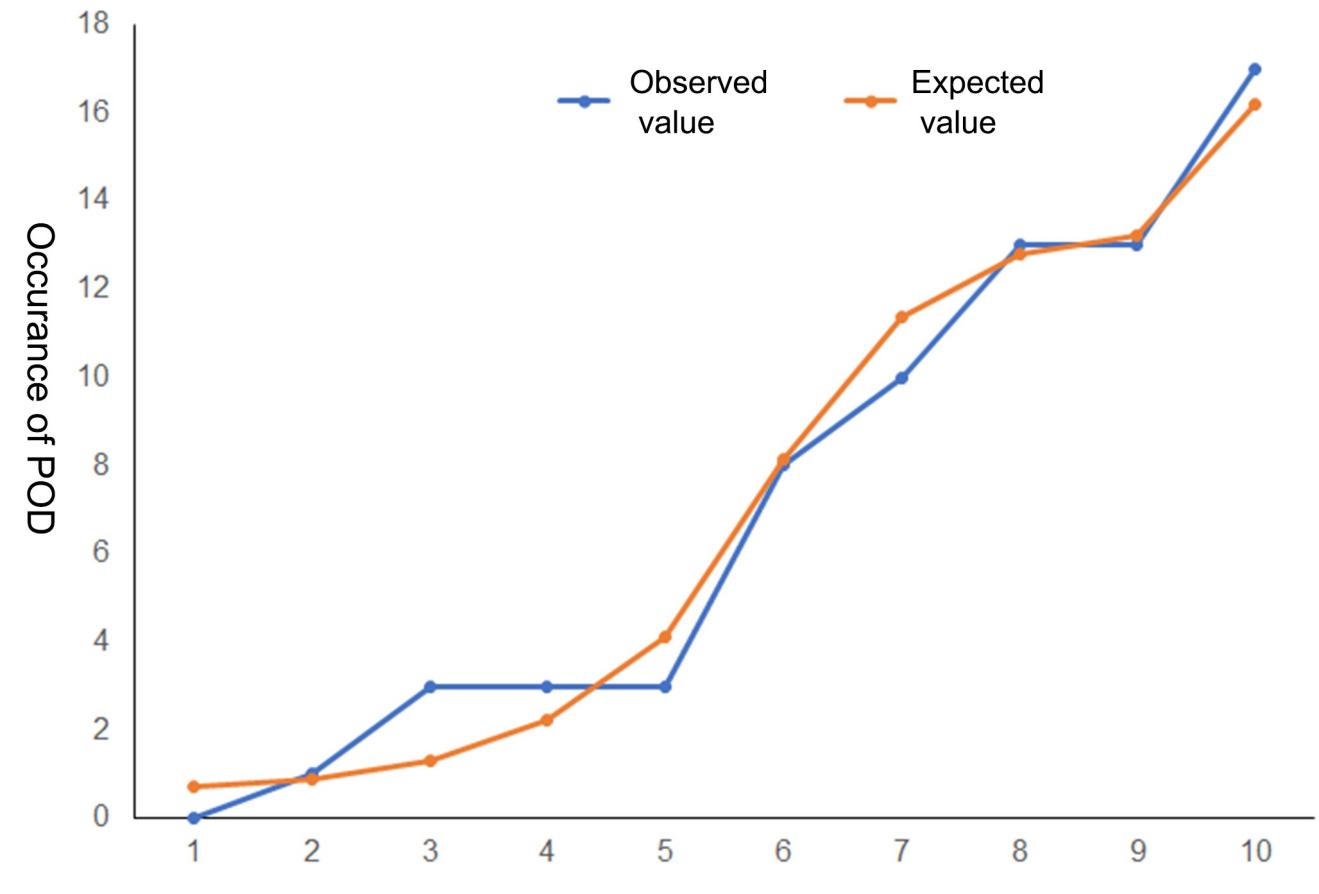
**

**Supplemental Figure 2.**  Calibration curve of a Modified composite score for predicting POD

**Supplemental document**

**DELi calculation**

EEG analysis and calculation software package (Beijing Easymonitor Technology Co., Ltd., Beijing, China) was used based on a wavelet algorithm, the most suitable tool for analyzing EEG. In the presence of delirium, repeatable and regular changes were extracted from brain waves as the characteristic indicators of objective cognitive function.

The theory of cognitive dynamic coordination is based on information interaction. Combined with EEG time-frequency and non-linear dynamic analyses, the EEG signals collected by scalp electrodes were decomposed into cortical and subcortical EEGs. The two-compartment model of “cortex and subcortex” was established. The static and dynamic EEG differences between left and right cerebral cortexes and subcortexes were calculated. The neural mechanism of the functional characteristics of the brain was explained from the angle of information interaction.

Left and right brain EEGs were sampled at 1,600 Hz with a sampling precision of 16 bits and a time window of 1 s. The EEG signals of the patients were collected through the EEG preamplifier and converted into discrete digital EEG signals after analog-to-digital (A-D) conversion. Discrete digital EEG signals were input into a computer and processed using a wavelet algorithm. In the process of noise reduction by decomposition and reconstruction, the coordination between dynamic and static components in time-space was calculated using multiple regression, power spectrum algorithm, pattern recognition and nonlinear dynamics. Thus, the aim of quantifying the features of brain neural function was achieved.

The dynamic coordination theory of cognition states that the speed difference of information transmission in the neural pathway correlates with the dynamic degree. However, the continuous closed-loop correlation formed by information transmission in the neural pathway correlates with the static degree and the dynamic and static coordination on the formation of a normal state of consciousness and awareness. During anesthesia, it was found that dynamic and static coordination would be broken, and dynamic and static components would change. At the temporal resolution, dynamic components decreased while static ones increased. At the spatial resolution, dynamic and static components changed. Based on the coordination change of temporal and spatial resolutions, the evaluation index of delirium based on EEG features was calculated.

If the $f\left( t \right)$function is a signal in the spatial domain$\left\{ -\infty,+\infty\right\}$, the scaling variable α takes control over the scaling of the wavelet function; the scaling variable τ takes control over the translation of the wavelet function; the scaling α is corresponding to frequency; the translation τ is corresponding to time:

$WT\left( \alpha,\tau\right)=\frac{1}{\sqrt{\alpha}}\int_{-\infty}^{+\infty} f\left( t \right)\psi^{*}(\frac{t-\tau}{\alpha})dt$ (1)

The basis function is a wavelet function in the form of:

$\psi_{\alpha,\tau}\left( t \right)=\frac{1}{\sqrt{\alpha}}\psi(\frac{t-\tau}{\alpha})$ (2)

A binary discrete wavelet transform was used for calculating the EEG vector. The formula is as follows:

$W_{2^{j}}f\left( k \right)=\left\langle f\left( t \right),\phi_{2^{j}}\left( k \right) \right\rangle=2^{-\frac{j}{2}}\int_{-\infty}^{+\infty} f\left( t \right)\phi^{*}\left( 2^{-j}t-k \right)dt$ (3)

Regarding the calculation formula of the wavelet frequency domain, the following expression was adopted:

$WT_{f}\left( \alpha,\tau\right)=\frac{\sqrt{\left| \alpha\right|}}{2\pi}\int_{-\infty}^{+\infty} F\left( \omega\right)\Psi\left( \alpha\omega\right)e^{j\omega\tau}d\omega$ (4)

Spectral analysis using the Fourier formula:

$F\left( \omega\right)=\int_{-\infty}^{+\infty} f\left( t \right)e^{-j\omega t}dt$ (5)

Inverse transform using:

$f\left( t \right)=\frac{1}{2\pi}\int_{-\infty}^{+\infty} F\left( \omega\right)e^{j\omega t}d\omega$ (6)

For EEG data, the present algorithm of wavelet analysis and the waveform reconstruction algorithm were used to process the particular EEG data vector, select the particular wavelet generating function and construct n scales and binary wavelet transform:

$\left( W\left( 2^{j},x \right) \right),j\in z$ (7)

The basis functions of the wavelet transform for a set of band-pass filters were obtained:

$\left( W\left( 2^{0},x \right) \right),\left( W\left( 2^{0},x \right) \right),\cdot\cdot\cdot\cdot\cdot\cdot\left( W\left( 2^{n},x \right) \right)$ (8)

Reconstruction was based on the reconstruction of the wavelet function and every wavelet basis function. The formulas are as follows:

$f_{j}\left( x \right)=\sum Wf\left( 2^{j},x \right)X2^{j\left( x \right)}$ (9)

$X2^{j(x)}$ represents the reconstructed wavelet, and *J* stands for the order of the time-domain function. Regarding the reconstruction of various wavelet basis functions, a set of reconstruction functions are as follows: $f_{1}\left( x \right),f_{2}\left( x \right)\cdot\cdot\cdot\cdot\cdot\cdot f_{n}\left( x \right)$; n = order.

The integral algorithm $f_{j}\left( x \right)$ was used for each waveform function obtained from the reconstruction of wavelet bases using the integral algorithm:

$WLE\left( i \right)={(\int f_{i}(x)dx)}^{2} ,i=1,2,\cdot\cdot\cdot\cdot\cdot\cdot n$ (10)

The power of the waveform potential $WLE\left( i \right)$ of every wavelet basis reconstruction function was obtained. The computational metadata of window EEG signals was obtained by a particular combination of computations:

$\{i\_10,i\_12,i\_15,i\_22,i\_23,i\_24,i\_35,i\_45,i\_48,i\_52,\ldots\ldots\}$ (11)

J represents the number of leads abbreviated as *i* series group among them, $\{i\_12,i\_15,i\_23,i\_45,i\_48,\ldots\ldots\}$ stands for the intermediate variable metadata of cortical EEG signals; $\{i\_22,i\_35,i\_60,i\_70,\ldots\ldots\}$ denotes the intermediate variable metadata of subcortical EEG signals; $\{i\_10,i\_24,i\_52,i\_66\ldots\ldots\}$ indicates the integrated intermediate variable metadata. $\left\{ i \mathrm{series}index group \right\}j=j\left\{ WLE\left( i \right),PSD\left( i \right),Gi\left( x \right) \right\}j\&\left\{ a1,a2,\cdot\cdot\cdot\cdot\cdot\cdot an \right\}j$ (12),$\left\{ a1,a2,a3,\ldots\ldots,an \right\}$ refers to the weighted coefficient of multivariate regression.

The decomposition and reconstruction of the above-mentioned metadata and the associations between different EEG signal leads were analyzed to obtain some cortical and subcortical intermediate EEG computing data units by clustering weighted calculation:

The dynamic component of the cortex is $D_{\mathrm{cor}}(j)=\{a_{1},a_{2},\cdot\cdot\cdot\cdot\cdot\cdot,a_{n}\}\&\{i\_12,i\_45,i\_48\}$ .

The static component of the cortex is $D_{\mathrm{sub}}(j)=\{a_{1},a_{2},\cdot\cdot\cdot\cdot\cdot\cdot,a_{n}\}\&\{i\_15,i\_23,i\_48\}$.

The dynamic component of the subcortex is $S_{\mathrm{cor}}(j)=\{a_{1},a_{2},\cdot\cdot\cdot\cdot\cdot\cdot,a_{n}\}\&\{i\_22,i\_60\}$.

The static component of the subcortex is$S_{\mathrm{sub}}(j)=\{a_{1},a_{2},\cdot\cdot\cdot\cdot\cdot\cdot,a_{n}\}\&\{i\_35,i\_70\}$.

The time series was set to $\left\{ x_{k}|k=1\cdot\cdot\cdot N \right\}$. N was set as the data length. In this case, $x(k)=(x_{k+\tau},x_{k+2\tau},\cdot\cdot\cdot,x_{k+(m-1)\tau})$. In the formula $k\leq N-(m-1)\tau$, $\tau$ represents time delay, and m stands for the number of embedded bits. A distance threshold δ was selected. For the initial point $x(0)$, the closest point to it was set as $x(k_{0})=Z_{0}(0)$,

After $t_{0}$ iterations, the distance between $Z_{0}(t_{0}) and x(t_{0})$ is greater than

δ, $l_{0}=\frac{1}{t_{0}}ln\frac{\left| \left| Z_{0}(t_{0})-x(t_{0}) \right| \right|}{\left| \left| Z_{0}(0)-x(0) \right| \right|}$. The point $x(k_{1})=Z_{1}(0)$ whose distance from $x(t_{0})$ is less than δ and the angle with $Z_{1}(0)-x(t_{0}) and Z_{0}(t_{0})-x(t_{0})$ is the smallest was taken. The process was repeated. It was assumed that until the end of step M-1,

$t_{M-1}=N-(m-1)$, then: $\lambda=\frac{1}{M}\sum_{k=0}^{M-1} l_{k}$.

($|D_{\mathrm{cor}}(j)-D_{\mathrm{sub}}(j)|$),($|D_{\mathrm{cor}}(j)-S_{\mathrm{cor}}(j)|$), ($|D_{\mathrm{sub}}(j)-S_{\mathrm{sub}}(j)|$) and (${|S}_{\mathrm{cor}}(j)-S_{\mathrm{sub}}(j)|$) were inserted into the above formula respectively. The final calculation:

$$DELi=e^{(\frac{D_{\mathrm{cor}}(j)}{\sum_{1}^{j} D_{\mathrm{cor}}(j)} / \frac{D_{\mathrm{sub}}(j)}{\sum_{1}^{j} D_{\mathrm{sub}}(j)} + \frac{S_{\mathrm{cor}}(j)}{\sum_{1}^{j} S_{\mathrm{cor}}(j)} /\frac{S_{\mathrm{sub}}(j)}{\sum_{1}^{j} S_{\mathrm{sub}}(j)} )}+e^{min(\lambda(|D_{\mathrm{cor}}(j)-D_{\mathrm{sub}}(j)|),\lambda(|D_{\mathrm{cor}}(j)-S_{\mathrm{cor}}(j)|),(|D_{\mathrm{sub}}(j)-S_{\mathrm{sub}}(j)|),\lambda({|S}_{\mathrm{cor}}(j)-S_{\mathrm{sub}}(j)|))}$$
